# The Importance of Alpaca Welfare in Human–Animal Interaction

**DOI:** 10.3390/ani16213481

**Published:** 2026-11-04

**Authors:** Monika Budzyńska, Joanna Kapustka

**Affiliations:** Department of Animal Ethology and Wildlife Management, University of Life Sciences in Lublin, ul. Akademicka 13, 20-950 Lublin, Poland; monika.budzynska@up.edu.pl

**Keywords:** alpaca, animal welfare, behaviour, handling, human–animal interaction, stress physiology, training, questionnaire

## Abstract

Human–animal interaction is considered one of the key determinants not only of animal welfare but also of the safety and ease of contact with humans. The aim of this paper is to review current knowledge on tools of assessment and improvement in human–animal interaction from an alpaca welfare perspective. Because alpaca-specific evidence is limited, the scientific evidence for some of the mechanisms and management strategies discussed is derived from other camelids or other animal species rather than directly from alpacas. Various factors affect the HAI quality, including early human contact, husbandry and veterinary procedures, handling, and training, as well as genetically influenced traits such as temperament variations. The methods used to evaluate the quality of HAI in alpacas mainly included three types of assessment tools: behaviour observation, measuring physiological parameters, and using questionnaires. Tools used to improve the quality of HAI in alpacas can include various techniques based on animal behaviour and learning knowledge, such as socialisation, desensitisation and operant conditioning. Considering human–animal interactions in alpaca husbandry (animals kept for fibre production, animal-assisted interventions or recreational activities) is crucial because it can ensure animal well-being, human safety and production/intervention effectiveness. Negative human–animal interactions can impair alpaca welfare through fear and stress as underlying mechanisms; however, practical applications (proper socialisation, handling, training) to achieve positive HAI and a positive perception of humans by alpacas could be better recognised and used, thereby benefiting their welfare.

## Simple Summary

Human–animal interaction strongly affects welfare, safety, use, and management. Positive experiences with humans, especially during early life, reduce fear and improve the human–animal relationship. Conversely, negative interactions can cause unnecessary stress and welfare problems. Nowadays, alpacas are used in various ways, not only for wool production, but also in animal-assisted intervention or recreation, which increases the frequency of contact with people. The aim of this review was to summarise current knowledge on human interactions with alpacas through assessment and improvement tools from an animal welfare perspective. It discusses welfare implications of human interactions and presents behavioural, physiological, and questionnaire-based methods used to assess the quality of these relationships in studies on alpacas. Based on scientific evidence from other species, interactions with humans can be improved by adapting animals to human contact, handling and training. Moreover, positive human–alpaca interactions may improve alpaca welfare, facilitate routine and veterinary procedures, and increase the safety of animals and caretakers. Further research is needed to identify practical indicators and strategies for improving human–alpaca relationships.

## 1. Introduction

**Figure 1:**
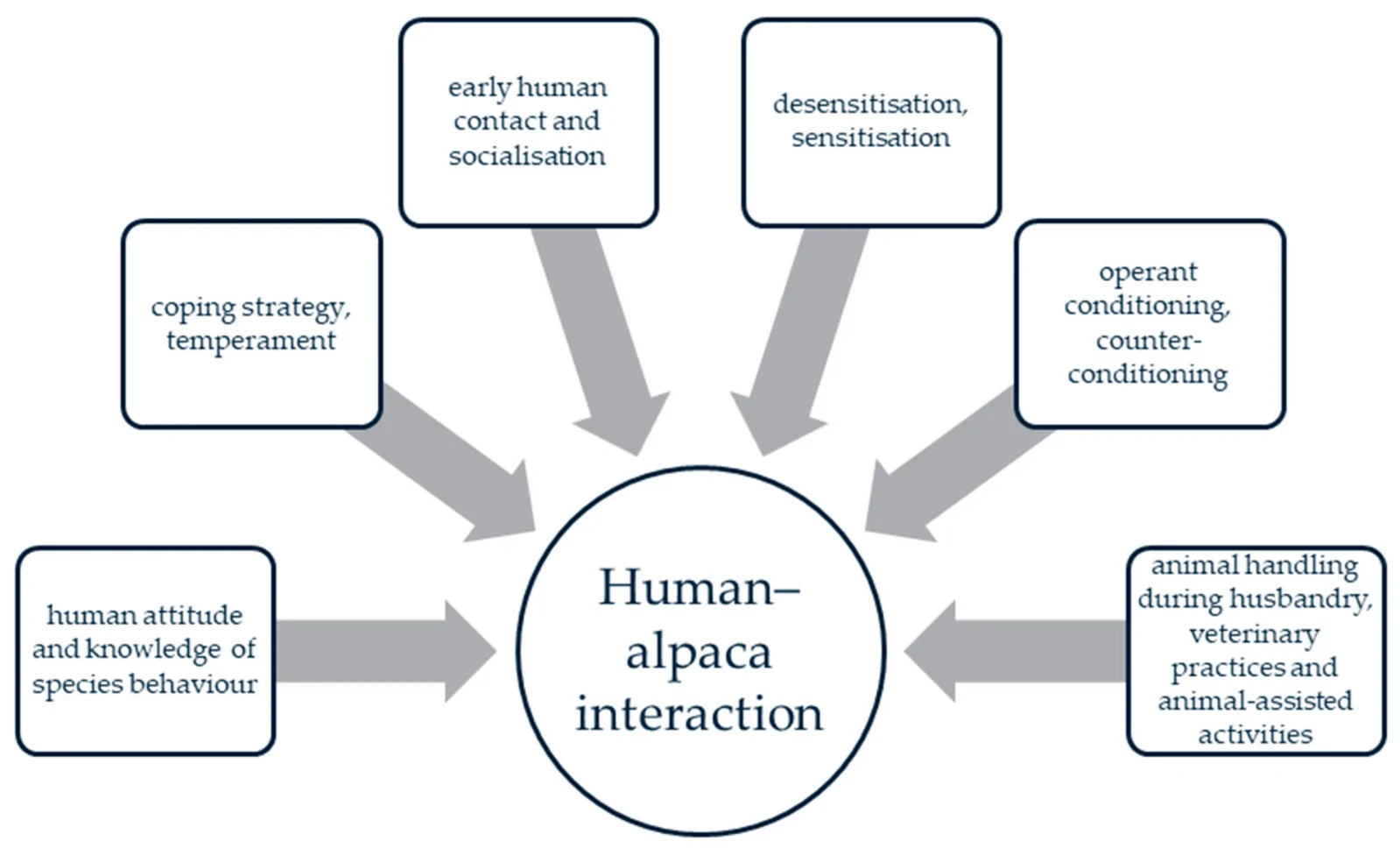
Potential factors and strategies that could influence the quality of human–animal interaction in alpacas.

Alpacas are one of four representatives of the South American Camelids (SACs). Alpacas and llamas are domesticated species, while guanacos and vicuñas are wild animals [1]. Alpacas are increasingly popular animals that are kept not only for high-quality fibre but also for animal-assisted intervention and agritourism [2]. Human–animal interaction (HAI) is considered a key determinant of not only animal welfare but also the safety and ease of contact with humans. Domesticated animals are not only exposed to human presence but also depend on people for survival, e.g., food and water access, shelter, etc. [3]. Alpacas should perceive interaction with humans as positive or neutral rather than as negative (stressful) when the animals fear people and avoid contact with them [4,5]. There are several factors affecting the quality of HAI. Research on HAI in alpacas has mainly focused on animal stress during various husbandry and veterinary procedures, but also on human perception of animal emotions as well as on the effects of caretaker attitudes and animal training [5,6,7,8,9,10,11]. Attitudes towards any animal species influence the quality of the human–animal relationship and the type of handling, and they can be classified as tranquil/friendly, dominant, impatient, and aggressive [12,13]. Animal response towards humans depends on the way of handling, and human activities including abrupt movement, pushing and shouting are considered negative handling, while activities based on slow movements, gentle petting and calm talking to the animals have friendly effects on animals [14]. Tools used to improve the quality of HAI in alpacas can include various techniques based on animal behaviour and learning knowledge such as socialisation, desensitisation and operant conditioning. Potential factors and strategies that influence human–animal interaction in alpacas are given in Figure 1.

The aim of this paper is to review current knowledge on tools for assessment and improvement in human–animal interaction from an alpaca welfare perspective. This review synthesises evidence on how to assess and improve these interactions. By focusing on behavioural, physiological, and questionnaire-based approaches, as well as factors affecting human–alpaca relationships, this review provides a focused perspective that complements existing welfare-oriented syntheses.

## 2. Methods

This narrative review was based on research and review papers published in scientific journals as well as books, book chapters, and conference contributions on human–animal interaction in alpacas and other representatives of SACs. The main types of research publications included original research articles and case reports that considered behavioural observation, measurement of physiological parameters, and questionnaire assessment, as well as studies combining these elements.

Because alpaca/other SAC-specific evidence is limited and the stated SAC-focused scope is broader in practice, we also considered evidence from other non-camelid species. This scientific literature search was conducted between March 2026 and July 2026, focusing on the digital databases: ScienceDirect, Scopus, Taylor & Francis and Web of Science. The search terms used in search were as follows (using logical operator OR): ‘alpaca welfare’ OR ‘alpaca behaviour’ OR ‘alpaca handling’ OR ‘alpaca training’ OR ‘alpaca learning’ OR ‘alpaca socialisation’ OR ‘alpaca human attitudes’ OR ‘alpaca stress physiology’ OR ‘camelids welfare’ OR ‘camelids behaviour’ OR ‘camelids training’ OR ‘farm animal welfare’ OR ‘human-animal questionnaire’ OR ‘human-animal survey’ OR ‘human-animal relationship’ OR ‘human-animal interaction’ OR, ‘environmental enrichment’. All terms were used in each digital database.

The search identified 15,264 articles before removing duplicate records and applying exclusion criteria (type of article, language of the article, relevance of the subject matter to the keywords). Approximately 60% of the retrieved articles were duplicates, resulting in a final dataset of 6105 records. Only scientific articles, books, book chapters, and conference reports were used in the review, which corresponded to the topic of the review. After introducing these criteria 100 articles were considered. Articles addressing topics outside the review scope and articles in languages other than English and Polish were rejected. Finally, 73 sources met the criteria.

As this study was designed as a narrative review, no standardised systematic-review procedure was employed. The retrieved publications were screened manually according to their relevance to the objectives of the review. In the first stage, titles were assessed against the predefined thematic scope and eligibility criteria. Records for which relevance could not be clearly established on the basis of the title alone underwent additional evaluation at the abstract level before a final inclusion decision was made. The review was divided into two main areas: (i) assessment tools in the field of human–alpaca interaction, including direct measures and questionnaire assessment of human–animal interaction, (ii) potential tools in HAI improvement, including early human contact and alpacas’ socialisation, practical use of learning processes in alpacas’ handling, and alpaca mental well-being in human–animal interaction.

## 3. Assessment Tools in the Field of Human–Alpaca Interaction

Assessing the quality of HAI in alpacas is an important way to measure and improve animal welfare. Assessment tools such as behavioural observations, physiological measures, and questionnaires have been used to gather and interpret data on HAI in alpacas. While questionnaires provide valuable information from the subjective perspective of HAI research participants, physiological and behavioural indicators measured in animals are important as objective, complementary tools to questionnaires [15]. Table 1 presents examples of some studies on HAI in alpacas and llamas, domesticated representatives of SACs. Behavioural and physiological responses recorded during procedures such as shearing or veterinary examination provide valuable information about animal stress and welfare in those specific contexts but they do not necessarily represent direct measures of the overall quality of the human–animal relationship. Assessment of animal–human interaction quality included direct measures of animals’ response to familiar and unfamiliar people in the animal enclosure or the pasture, as well as assessments of owners’ attitudes toward animals based on questionnaire studies.

### 3.1. Direct Measures of Human–Animal Interaction

The level of human impact on an alpaca can be measured by direct contact in neutral conditions (e.g., at pasture) and also by an animal’s response during routine husbandry procedures (e.g., during shearing) [17]. Behavioural assessment of HAI can involve indicators known as proximity to humans or flight distance. Proximity to humans was used as a behavioural indicator of HAI in llamas, and it was evaluated as the number of animals in the presence of a familiar or unfamiliar person [16]. Alpacas and llamas, like other prey animals, e.g., cattle [24], also show so-called avoidance distance (AD) or flight distance (FD). FD can be described as the minimum space between an approaching person and an animal beyond which the animal runs away [25]. Animal reaction to human presence can also be considered a significant temperament trait [25]. To our knowledge, the first study on domesticated SACs’ responses to human contact used proximity to humans as an indicator of HAI (measured in an experimental outdoor enclosure). It revealed that llamas can distinguish individual humans and react negatively to unfamiliar people [26]. The authors also note that llamas may discriminate against humans involved in past aversive procedures, which can increase the risk of more difficult future interactions, especially if relevant training is not provided. Thus, considering these effects is important in scheduling animal caregivers, planning research design and developing handling and training programmes for animals [16]. In contrast, research on alpacas did not find that a shorter flight distance from humans (measured during human approach on pasture) correlated with a lower stress response during shearing [17]. However, the animals with a shorter flight distance from a familiar person at pasture also showed a shorter FD from an unfamiliar one. It was concluded that individual characteristics of alpacas can shape their responses to humans (familiar and unfamiliar persons), and considering the alpacas’ predispositions for their use in AAIs and agritourism is especially important [17]. Behavioural observation also includes monitoring animal responses such as stress-related behaviours: flight attempts, kicking, spitting, and vocalising during various husbandry (e.g., shearing, toe trimming) and veterinary (e.g., blood sample collection, disease treatment) practices in alpacas [8,11,17,21]. Humans may perceive these approaches as threatening; however, animals may respond with escape or freezing [27]. It is known that stressful situations can elicit different coping strategies in individuals of the same species, as shown, for e.g., in horses and camels as well as in alpacas [8,17,28,29]. A proactive strategy is shown by higher activity of the sympathetic–adrenal medulla (SAM) axis and active defence behaviour, while a reactive strategy is associated with higher activity of the hypothalamic–pituitary–adrenal cortex (HPA) axis and passive behaviour [28]. It was found that alpacas can differ in their behavioural and physiological reactivity during human presence in various situations. The animals with shorter FD (measured in neutral conditions—at pasture) were more willing to reveal active defence responses (abrupt movements and vocalisations) during shearing. In comparison, alpacas with longer FD showed a tendency to passive reactions (freezing behaviour) to shearing stress [17]. Moreover, a negative correlation between alpacas’ plasma cortisol concentration and vocalising frequency during shearing was found, which implies that intense vocalisation is characteristic of a proactive type of strategy to cope with stressful situations [8]. The study of Kiene et al. [20] showed that freezing and collapsing were associated with higher serum cortisol and plasma glucose levels during clinical examination in alpacas, while spitting, kicking and screaming did not correlate with plasma glucose levels. Stress signs such as freezing and collapsing can be recognised as responses characteristic of a reactive coping strategy, and defensive behaviours such as spitting, kicking and screaming can be recognised as typical of a proactive strategy. Observed a few days after shearing, a positive relationship between plasma noradrenaline levels and standing and grooming behaviour can also suggest a tendency toward a proactive coping strategy, where higher activity of the SAM axis involves active behavioural responses [8]. Assessment of alpacas’ stress during shearing with the use of various methods of restraint (standing up, lying down or on the table) showed an increase in faecal cortisol metabolites in animals of all restraint groups; however, salivary cortisol level increased only in the animals sheared on the ground [9]. Later studies on shearing stress also confirmed the increase of salivary cortisol in alpacas in lying position on the ground [17,18] and no increased plasma cortisol in alpacas sheared in standing position was found [8]. Moreover, behavioural assessment showed that fewer alpacas vocalised when sheared standing up [11]. These findings suggest that the standing position is less stressful for tamed animals; however, individuals showing intense defence behaviour are suggested to be sheared either lying on the ground or on the table to decrease the injury risk [9]. Further research in this area could be useful for establishing clear criteria for selecting a restraint method with regard to individual animal behaviour and safety considerations.

The reaction to human presence can also be assessed during procedures connected with physical and veterinary examination as well as disease treatment. Veterinary practices are recognised as stressful, and factors such as the presence of unfamiliar persons, capture, restraining, handling alone, and medical care are indicated as common stress agents [10,21]. Monitoring disease progression and stress responses in alpacas with dermatological problems revealed increased vigilance, vocalising, jumping, running, and tachycardia and tachypnoea [21]. Moreover, fear and pain connected with veterinary interventions and treatment may even worsen the animal’s disease [30,31]. Understanding and recognising stress in alpacas is crucial in veterinary practices for effective disease treatment and these animals’ well-being [21]. Alpacas’ stress response to handling during veterinary examination (health check, intramuscular vitamin supplementation, rectal faecal sample collection) was measured by thermal imaging and a rise in surface body temperature was noticed [22]. However, these authors also confirmed no significant increase in salivary cortisol level in alpacas before and after veterinary examination. Husbandry and veterinary practices also include health and body condition score (BCS) assessments, which can be important tools for evaluating alpaca welfare. Alpacas did not show many severe stress behaviours during physical examination (BCS scoring and health check), but handling problems occurred when animals were introduced to the examination box, and anxiety signals were observed in their body language [5]. Moreover, there were some negative correlations between stress-related behaviour (spitting) and health indicators (BCS, eye mucous membrane colour) that can suggest a potential association between alpaca stress and health [5].

### 3.2. Questionnaire Assessment of Human–Animal Interaction

Questionnaires provide significant information about the personal experience of HAI study human participants and the animals in their environment [15]. Based on alpaca caretakers’ survey, it was found that gentle tactile, auditory and visual handling is related to easier interaction during moving and leading and a higher probability that an animal would rather approach its handler than move away [23]. This study also suggested that non-aversive training can improve the ease of alpacas’ handling. Moreover, early gentle handling (e.g., frequent stroking of young animals and talking to them) has beneficial effects related to easier handling in later life [10]. It was also stated that negative general caretakers’ attitudes were correlated positively with the occurrence of behaviour problems [23]. On the other hand, evaluated in the alpaca caretaker’s survey, positive features of the animal such as adaptability to novel situations and gentleness were negatively associated with the duration of the physical examination by a person unfamiliar with the animal, while the aggression trait was associated with kicking behaviour [5]. A caretaker’s questionnaire assessment of the animal’s behaviour traits is a subjective tool; however, it is based on everyday presence during animal handling [32,33]. Knowledge of body language provides important information to predict habitual or sporadic animal responses to human presence, and particularly ear positions, but also their combinations with head and neck positions, are significant indicators of positive or negative HAI [34]. For example, a positive emotional response to humans could be recognised by signals of curiosity and interest, when an alpaca’s ears are raised and pointed toward the human, a neck and body form an angle of 90 degrees, and a loosely lowered tail is observed [7]. On the other hand, different signals can be manifested by anxious or submissive alpacas, having the ears laid back, the front part of the body lowered, and the tail slightly raised. While the warning posture (before defensive aggression with spitting) is characterised by the ears laid back, the head held high and the tail in a horizontal position [7]. Respondents to the questionnaire on body language interpretation had some difficulties with proper recognition of alpacas’ signals, and especially alert, warning and offensive aggression postures were correctly interpreted by the lowest number of survey participants [7]. A lack of familiarity with and correct interpretation of—specifically—the warning posture (against spitting) increases the risk of being spat on; while this may not be dangerous, it is generally perceived negatively. Additionally, when an animal sends such a signal, it means a human has crossed its comfort zone. From the perspective of human–animal interaction, this situation is negative for both sides. Recognising warning signals in alpacas is very important because defensive and offensive aggression can include biting, bumping, or kicking, and these kinds of responses can put both alpacas and handlers in danger [10,35]. Furthermore, a frightened animal will avoid contact with humans, and the establishment of a positive interaction between man and animal without alleviation of fear is not possible [7].

## 4. Potential Tools in HAI Improvement

Several strategies can be used to improve human–alpaca interaction, including early contact with humans, various training methods, as well as guidelines from the animal welfare perspective based on species behaviour knowledge. For example, training techniques that rely on positive reinforcement have been used for handling, shearing, veterinary procedures, and also loading and unloading animals for transport [35]. In domesticated animals (e.g., horses), the process of building a human–animal relationship begins immediately after birth [36], or even earlier. The phenomenon, called vertical social transmission, is based on acquiring information from parents [37]. In horses, foals whose mothers remained calm while being groomed and fed by humans were more willing to stay close to people and engage in interactions with them (such as sniffing or licking) [38]. However, this phenomenon has not been thoroughly investigated in animals other than horses. No such relationship has been demonstrated in cows [37], but the authors emphasise the need for further research in this direction. Other well-known directions include animals’ socialisation [39] and handling based on learning processes that build trust and a sense of safety [40]. Moreover, an appropriate level of mental well-being should be ensured for both animals kept solely on farms and working animals [41].

### 4.1. Early Human Contact and Alpacas’ Socialisation

As a very broad concept, socialisation of animals covers many issues and processes. It starts in early life and is essential for young organisms to survive. Social animals need to learn the norms of their species [42]. As farm animals, alpacas, especially those kept in small herds, are in almost constant contact with humans. Therefore, we can distinguish between intra-species and inter-species socialisation, which is observed in different farm species [12].

The use of sensitive periods in early life socialisation seems to be beneficial [43]. Sensitive periods are defined as short periods in early life when the animal is more susceptible to new stimuli [44]. In farm animals, the period of sensitivity is discussed in the context of its use in establishing relationships with humans, which in the future is expected to result in less anxiety, easier handling and higher productivity [39]. Initially, this process is necessary to establish a bond, especially with the mother, and to instil the behavioural norms of a given species [42]. The time of its occurrence depends on the species. In lambs, the first bond with the mother develops within two hours after birth [45]. During this time, contact with the mother should be undisturbed. In the first weeks of life, this process progresses [44]. There is no evidence about alpacas or other camelids; therefore, the specific nature of this phenomenon in this species, and whether it shares characteristics with the examples cited here, remains a matter of hypothesis. Young organisms learn social hierarchy and species-specific norms, which is especially important for forming bonds with conspecifics [42]. After birth, the alpaca female does not lick or groom the cria, but sniffs it and communicates with low-frequency vocalisation, which the cria responds to in the same way [46]. The mother will protect her offspring and spit on the other crias when they try to milk her; some evidence of allosuckling behaviours occurs [47]. In alpacas, crias are the lowest in the hierarchy pyramid [48]. Immature alpacas show a submissive crouch position in relation to mature conspecifics [7]. A playing behaviour acts an important role in social development in lambs and calves, which increases in frequency when kept in groups [49,50], and may also be considered desirable in alpacas.

Socialisation is mostly based on the habituation process, defined as a decrease in response to a stimulus that is regularly repeated without reinforcement or other consequences [25]. This means that if any stimulus (e.g., sounds of cars on the road) is repeated many times and nothing happens (no reward or punishment), the animals will stop reacting to it. There is a need to differentiate habituation from adaptation or fatigue. In animals’ socialisation, long-term habituation is needed. The lack of reaction must be constant over time, not momentary [51]. The technique that uses the habituation process deliberately and in practice is called desensitisation [40]. Alpacas are naturally neophobic and react to new stimuli more with fear than curiosity [52]. Desensitisation involves deliberately and gradually exposing an animal to a stimulus (e.g., touching the neck) to eliminate the initial fear response (e.g., neck retraction). The most important thing is a gradual increase in the intensity of a stimulus when there is no response to the previous one [53]. Desensitisation, especially the tactile one, is one of the elements of both early and later socialisation of alpacas.

For many years, alpacas’ owners and breeders were afraid to touch or induce any other interaction with crias, because of the risk of Berserk Male Syndrome (BMS). This disorder is defined as over-socialisation with humans, resulting in the inability to function properly in the herd in adult life. The higher risk factor is bottle-feeding, but it is not the only one [34,54]. Adult alpacas with the BMS behave aggressively toward humans and do not know how to behave properly toward conspecifics. Sometimes the only solution is euthanasia [54]. There is a real problem, but not as common as it might seem. The research on other farm species (calves, lambs) shows that early socialisation (gentle positive contact) reduces fear of stockpersons and makes handling easier [50,55,56]. The studies of Waiblinger et al. [11] and Windschnurer et al. [10,23] gave evidence for alpacas. They show that young alpacas and llamas who experienced gentle positive touch contact with a human as a cria were calmer in the future and less willing to baulk during procedures. This result is consistent with observations in other farm animal species [50,55,56]. Both excessive socialisation with humans and the lack thereof are unfavourable. Gentle tactile contact with cria, not too excessive, can be considered as the first part of human–alpaca socialisation.

This process does not only refer to building relationships with humans but has a broader context. An enriched environment in early life (in farm animals: pigs, cattle and llamas), like a bigger area [57,58], contact with unfamiliar and familiar conspecifics [57], access to toys [58,59] and grooming tools (brushes) [26], increases social, exploratory and playing behaviours [26,58], cognitive abilities [57] and reduces animal stress [59]. Enrichments for adult animals are also beneficial. The latest study on llamas [60] shows that the best enrichment for them was PVC tubes filled with hay or elephant grass. They were not very interested in tyres, brushes or balls, but the authors admitted that the study was conducted on a small sample. It would be interesting to repeat the test. Alpacas and llamas are herbivores; due to their origin, it is natural for them not only to graze, but also to browse their most preferred forage [48]. Another type of enrichment for llamas and alpacas can be cognitive-social enrichment. In the study of Pahl et al. [3], llamas (accustomed to people) observed humans or other llamas solving a problem (bypassing a barrier and obtaining food). Llamas were more successful after demonstrations by both humans and conspecifics, but they did not copy the behaviour; they understood the solution. For herd animals, other individuals (both animals and humans) can be a useful source of information.

Socialisation is a process that occurs throughout an animal’s life. It occurs most effectively in early life [11,50,55,56], being a first step in building a human–animal relationship, but it does not end with maturity. Alpaca owners and breeders should balance risk management (e.g., BMS) with the benefits of appropriate socialisation practices in early life.

### 4.2. Practical Use of Learning Processes in Alpacas’ Handling

Young alpacas are usually weaned at the age of 6 months, and females come back to the mother’s herd after four to six months. Weaning is a stressful experience for both the mother and her offspring [42]. Young alpacas need to adapt to this new situation before the structured training is started. The effectiveness of training increases if the youngster has previously been socialised with humans [11,43] and a bond has been created between them [41]. In horses, the process of socialisation with humans serves primarily to prepare the animal for training and handling. It starts in the first hours of life but must be minimal and gradually increased in the following weeks and months. After weaning, the animal starts to be more independent, and the trainability increases [36]. A similar pattern may apply to alpacas.

McGreevy et al. [61] claim that the relationship between an animal and a trainer or owner should be based on trust and confidence, but these terms are not fully defined and difficult to study scientifically. This effect is likely driven by decreased fearfulness combined with a pronounced positive response to human interaction [61]. However, Ramos and Williams [62] conducted an in-depth interview with the marine mammals’ trainers and observed that the human–animal bond and trust can be measured. The human–animal bond is interpreted as a cognitive, emotional and behavioural relationship based on mutual interaction and influence in long-term contact. The approach to interaction and training must be individual, taking into account personality, preferences and cognitive differences. Trust between an animal and trainer is built on positive experiences (incl. positive reinforcement in training), emotional closeness and mutual attachment. Alpacas are herbivores living in a herd, characterised by neophobia, vigilance, and a flight reaction when feeling threatened, typical for prey animals [34,63]. Therefore, they differ significantly from marine mammals, but the basis of the learning process and conditioning is the same for all mammals.

**Figure 2:**
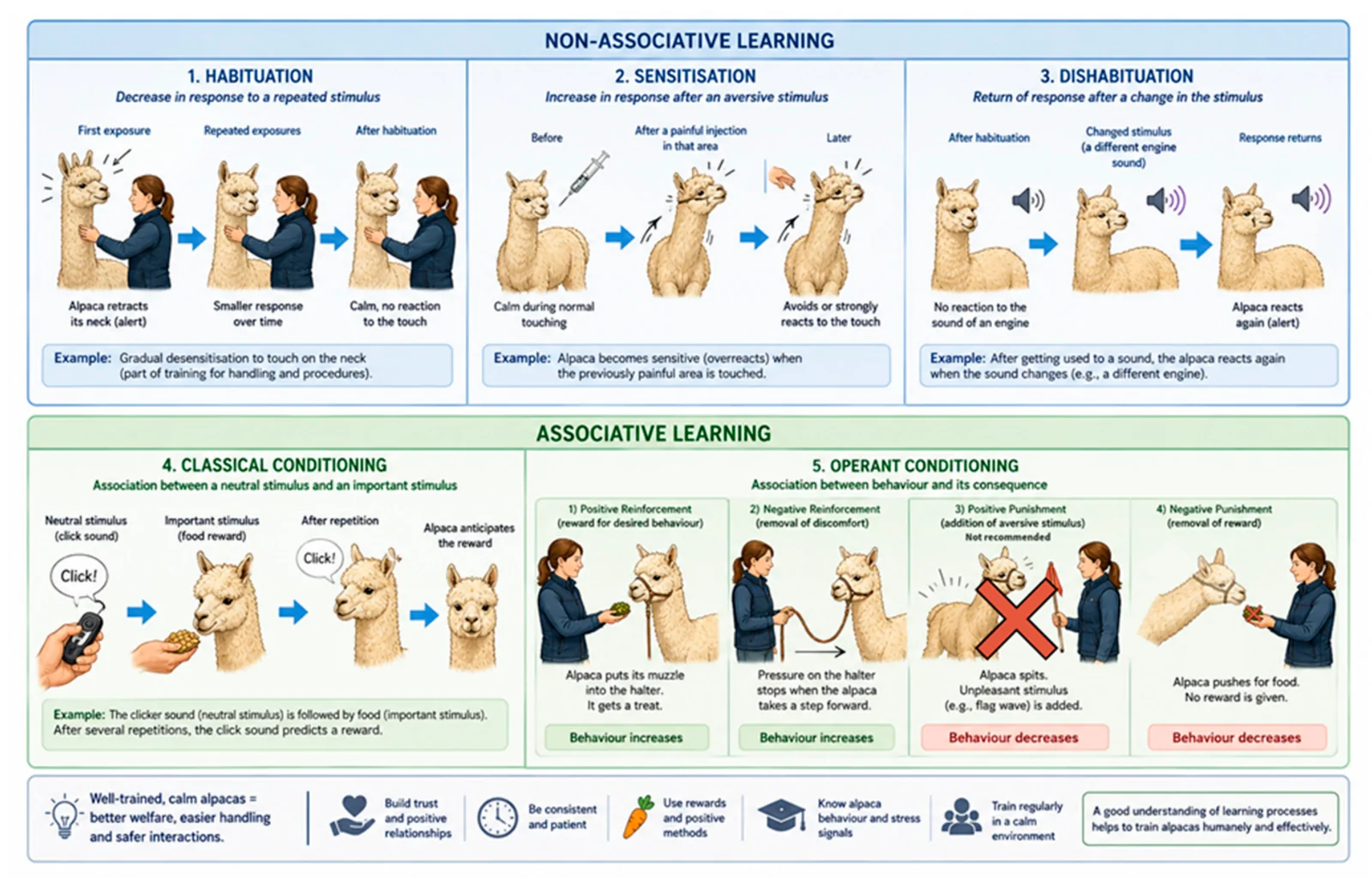
A proposed conceptual model of learning processes that could be used in practice, in alpaca training and handling, based on scientific evidence [40,51,53] (figure created with the help of generative AI, prompt written by the authors).

Alpacas have been bred for centuries as wool-producing animals, kept extensively in large herds on high-altitude Andean pastures [64]. Primarily, they were not accustomed to frequent and close contact with humans. Most publications on alpaca training and bonding come from the United States [65] and Europe [6,10,23]. Alpaca training can be based on specialised approaches, such as Camelidynamics [65], which primarily rely on desensitisation techniques, and operant conditioning (e.g., clicker training, positive and negative reinforcement) [6,40]. As mentioned in the previous paragraph, desensitisation is based on the habituation process. This is a reversible learning process. As a result of a change in the stimulus’ characteristics, the response to it may return (this is called dishabituation) [28]. The structured training starts with learning halter acceptance [6,65] through desensitisation [40,51] and walking on a lead rope [6,65], based on negative reinforcement [40]. The Camelidynamics method, based on, described above, learning techniques, was also the subject of the research on alpacas’ behavioural reactivity during training [6]. The study by Windschnurer et al. [23] reported an association between the use of Camelidynamics and improved ease of handling in alpacas. Camelidynamics is not the only method for working with alpacas. Training using a clicker or simply positive reinforcement (desired behaviour = reward) can also be used [6,40]. The choice of method should be tailored to the individual animal’s characteristics and one’s own experience. Learning processes, such as habituation, classical and operant conditioning, are widely used in animal training, in both practical and research contexts [23,40,51]. In the next steps, the alpacas are taught to give their legs for toenail trimming and accustomed to husbandry and veterinary procedures (drug administration, examination performance (e.g., ultrasound), inspecting and palpating body parts) [54] via desensitisation or counterconditioning [40]. These procedures can be performed with very little or no stress if previously trained. Negative experiences, such as a painful injection, can increase an animal’s defensive response and sensitise its specific body area to human touch. This learning process is called sensitisation [28]. Counterconditioning is helpful when an animal reacts excessively negatively to a stimulus (e.g., it does not allow itself to be touched in the area where a painful injection was administered). It involves pairing a positive stimulus (e.g., food) with a negative one (touching an unacceptable area or its vicinity). It is important to reward the animal only when there is no negative reaction [40,53]. For decreasing undesired behaviours, positive and negative punishments are used. In learning theory terminology, “positive” refers to the addition of a stimulus—for example, positive punishment involves adding something unpleasant to the environment—while “negative” refers to the removal of something from the environment—for instance, negative punishment involves taking away something desirable [40]. Positive punishment, such as pinching and kicking by humans or intimidation by waving a small flag, negatively affects the relationship with the animal and should not be used. Negative punishment is used to extinguish undesirable behaviours (e.g., when an alpaca forces food and does not get it, it stops doing it after a while) (Figure 2). The learning types that could be used in alpaca training and handling are given in Figure 2.

### 4.3. Alpaca Mental Well-Being in Human–Animal Interaction

Animal welfare is one of the key issues in alpaca husbandry. The human–animal relationship is an integral part of it because humans are responsible for all aspects of animal welfare, from feeding to preventive health care [5]. Many authors underline [27,66,67] that positive or negative interactions with humans strongly affect animal welfare. In dairy cows, negative handling behaviours include slaps, hits and tail twisting. The stockpersons rated the cows as unpleasant and difficult to handle. This resulted in increased fear of humans and decreased milk production [27]. Similar observations were made by Windschnurer et al. [23] on alpacas and llamas. On farms, where animals were more difficult to handle, owners also held a negative attitude toward their animals. This phenomenon also depends on other factors, such as the stockperson’s technical knowledge, nutritional deficiencies or even genotype [68]. It is often challenging to disentangle whether problematic behaviour in animals is caused by inadequate treatment or whether the animal’s inherent characteristics influence the handler’s negative perception. Negative human–animal relationships are likely influenced by multiple factors, and each case should therefore be assessed individually. There are a number of factors that can contribute to alpacas’ stress during HAI, such as the presence of familiar and unfamiliar persons during various routine husbandry and veterinary procedures, as well as animals’ contact with unknown humans during animal-assisted interventions (AAIs). Some studies on the quality of HAI in alpacas measured during both everyday human contact and some procedures have been reviewed in the second section of this paper. However, while the benefits of alpaca-assisted interventions for humans are known, there are no data available concerning the potential effects the AAIs may have on the alpacas [69,70,71]. During animals’ participation in this specific kind of HAI, they can experience discomfort, as well as physical and mental stress, so it is crucial to ensure animal health and welfare in all aspects of AAIs [72]. Alpacas’ suitability to participate in therapy and recreational activities (e.g., walking, trekking) should be based on animal predispositions, including their mild temperament and training ability [6]. Alpacas should be adapted to strangers, frequent touch and should be able to accept a halter and walk on a leash. They should also be familiar with being transported in a car or a livestock trailer [6].

It is important to underline that stronger emphasis should be placed on building a positive human–animal relationship rather than finding those responsible for this wrong one. Rault et al. [67] highlight that positive human–animal interaction is a powerful tool in animal husbandry. It is not only a lack of fear but also that the animal voluntarily approaches and seeks contact with humans, showing relaxation and pleasure [67]. This concept aligns closely with the modern interpretation of the Five Freedoms, which emphasises that animal living conditions should not merely meet minimum standards but should be optimised to ensure a life worth living. The term welfare of an animal should be understood not only as the absence of negative states, such as fear, but also as a condition in which positive experiences outweigh negative ones [41]. An approach based on building relationships and trust also contributes to increased welfare, easier handling and safety [61].

People interact with alpacas in various places and situations. Some information exists on the conditions of keeping alpacas on farms in the context of their welfare [5,34,60]. However, in Europe, many alpacas are transported to kindergartens, schools, local picnics or festivals, or for walks. People visit farms to spend time with alpacas. Therefore, alpacas used as recreational and AAI animals experience novel environments and humans, which can contribute to animal stress. For these uses, a new welfare protocol for working animals should be developed. Below are some guidelines from the animal welfare perspective based on alpaca behaviour and biology knowledge:

- As herd animals, alpacas should never be kept alone or separated from their herd members [5,34,73]
- As herbivores, alpacas’ working hours should not disrupt their time budget, especially the time spent on food intake (ca. 40% of overall daily activity) [73]
- As prey animals, alpacas should be desensitised and trained to accept the proximity of strangers. People should not surround alpacas, hug (especially embracing the head) or wave their arms, as this could be perceived as a predatory attack [6,54]
- As mammals, crias (young alpacas) should not be separated from their mothers until they are at least 6 months old [34]
- In the context of the human–animal relationship, the owner should create a strong bond with their animals based on trust and a feeling of safety [10,23,67]
- The owners should have advanced knowledge about alpacas’ behaviour, especially stress signals, like moaning, spitting, and escape attempts [7,34].

Although these few tips can be considered practical recommendations for the welfare protocol for working alpacas in the context of human interaction, they constitute an important basis that can facilitate the use of alpacas in this direction while ensuring an appropriate level of welfare.

## 5. Conclusions and Future Directions

Considering human–animal interactions in alpaca husbandry (animals kept for fibre production, animal-assisted interventions or recreational activities) is crucial because it can ensure animal well-being, human safety and production/intervention effectiveness. Negative human–animal interactions can impair alpaca welfare through fear and stress; however, practical applications to achieve positive HAI and a positive perception of humans by alpacas could be better recognised and utilised, thereby benefiting their welfare. Human–alpaca interaction can be improved by animals adapting to human presence through proper bond development based on alpacas’ socialisation and the use of learning processes in alpacas’ handling and training. However, as the review indicates, positive effects depend on the timing, quality, predictability, and appropriateness of this interaction. Based on our review of the alpacas’ scientific literature, there is still a limited number of papers on the assessment of their behavioural and physiological parameters from a welfare perspective. Therefore, we also considered evidence from other animal species (other camelids and non-camelid species). In the field of human–alpaca relationships, both direct measures (behavioural observations and physiological indicators) and questionnaire tools can be used to assess the quality of HAI regarding everyday handling as well as different husbandry and veterinary practices. Future research is needed on factors affecting HAI in alpacas, welfare indicators to assess HAI quality that can be used by animal owners, producers and veterinarians, as well as the welfare-friendly techniques that can improve handling procedures in these animals. In particular, specific research priorities emerging from this review can include validation of alpaca-specific HAI indicators, appropriate socialisation and training strategies, differentiation between acute procedural stress and HAI quality, and systematic welfare assessment of alpacas involved in animal-assisted interventions.

## Figures and Tables

**Table 1:** Examples of HAI assessment in domesticated SACs.

HAI Dimension Assessed	SACs Species (Animals’ Number or Questionnaire Respondents’ Number, Type of Article)	Assessment Tools	References
animal–human interaction quality: assessment of responses to familiar and unfamiliar person	llama, *n* = 5, animal-level intervention trial [16], alpaca, *n* = 34, animal-level intervention trial [17]	behavioural observation (proximity to humans, flight distance from humans)	[16,17]
acute stress during human contact: assessment of responses to shearing procedure	alpaca, *n* = 20, animal-level intervention trial [8], *n* = 60, animal-level intervention trial [9], *n* = 60 animal-level intervention trial [11], *n* = 34, animal-level intervention trial [17], *n* = 22, animal-level intervention trial [18], *n* = 58, animal-level intervention trial [19], *n* = 125, animal-level intervention trial [20]	behavioural observation (escape attempts, spitting, vocalising), physiological measures (cortisol, dopamine, noradrenaline, serotonin, thyroxine in blood, faecal cortisol metabolites, salivary cortisol, body temperature, heart rate, respiratory rate)	[8,9,11,17,18,19,20]
acute stress during human contact: assessment of responses during physical, veterinary examination and treatment	alpaca, *n* = 30, animal-level intervention trial [5], alpaca and llama, *n* = 116, animal-level intervention trial [10], alpaca, *n* = 125, animal-level intervention trial [20], alpaca, *n* = 4, case report [21], alpaca, *n* = 8, animal-level intervention trial [22]	behavioural observation (e.g., body language, collapsing, freezing, kicking, spitting, vocalising), physiological measures (e.g., salivary and serum cortisol, plasma glucose, surface body temperature)	[5,10,20,21,22]
animal–human interaction quality: assessment of owners’ attitudes toward animals	respondents that kept alpacas, *n* = 30, questionnaire [5], *n* = 157, questionnaire [23], respondents that kept both alpacas and llamas, *n* = 116, questionnaire [10], *n* = 27, questionnaire [23]	questionnaire (e.g., animal behaviour towards its caretaker’s approach, ease of handling in various situations, behaviour problems from the caretaker’s point of view)	[5,10,23]
animal–human interaction quality: assessment of human ability of animal body language recognition	alpaca, *n* = 103, questionnaire [7]	questionnaire (interpretation of alpaca body language presented in the questionnaire images: pictures of alpacas with different body postures)	[7]

## Data Availability

Available upon reasonable request.

## Abbreviations

The following abbreviations are used in this manuscript:

SACs	South American Camelids
HAI	Human–Animal Interaction
BCS	Body Condition Score
BMS	Berserk Male Syndrome
AAIz	Animal-Assisted Interventions
FD	Flight Distance
SAM	Sympathetic–Adrenal Medulla
HPA	Hypothalamic–Pituitary–Adrenal
